# Endoplasmic Reticulum Stress Coping Mechanisms and Lifespan Regulation in Health and Diseases

**DOI:** 10.3389/fcell.2019.00084

**Published:** 2019-05-21

**Authors:** Sarah R. Chadwick, Patrick Lajoie

**Affiliations:** Department of Anatomy and Cell Biology, The University of Western Ontario, London, ON, Canada

**Keywords:** endoplasm reticulum stress, aging, neurodegeneration, unfolded protein response, proteostasis

## Abstract

Multiple factors lead to proteostatic perturbations, often resulting in the aberrant accumulation of toxic misfolded proteins. Cells, from yeast to humans, can respond to sudden accumulation of secretory proteins within the endoplasmic reticulum (ER) through pathways such as the Unfolded Protein Response (UPR). The ability of cells to adapt the ER folding environment to the misfolded protein burden ultimately dictates cell fate. The aging process is a particularly important modifier of the proteostasis network; as cells age, both their ability to maintain this balance in protein folding/degradation and their ability to respond to insults in these pathways can break down, a common element of age-related diseases (including neurodegenerative diseases). ER stress coping mechanisms are central to lifespan regulation under both normal and disease states. In this review, we give a brief overview of the role of ER stress response pathways in age-dependent neurodegeneration.

## Introduction

Protein homeostasis (or proteostasis) is the sum of cellular processes involving protein transcription, translation, folding, and degradation (Balch et al., 2008). In order for a cell to remain functional and capable of adapting to changing biochemical and environmental signals, proteostasis must remain uncompromised (Ben-Zvi et al., 2009). Protein folding is particularly important for cellular processes, as the final conformation of a folded protein is essential to its function. Cellular membrane dynamics are a pivotal aspect of protein folding; adaptations in the ER membrane’s composition and size are required to maintain proteostasis, and proper protein folding, in turn, is required to maintain this membrane integrity (Hou and Taubert, 2014).

Under normal circumstances, proteins destined for the secretory pathway are translated directly into the ER via ribosomes embedded in the ER membrane, bound by chaperone proteins, folded, and then packaged into vesicles for secretion (Novick et al., 1981). This includes proteins destined for the plasma membrane, such as membrane-linked receptors, or secreted factors released into the extracellular environment. In some cases, however, this pathway can go awry; proteins may become misfolded or unfolded in the ER, and unable to be recovered by the protein quality control machinery. In this instance, the improperly folded protein is targeted for degradation, exported into the cytosol, and degraded by a proteasome (Werner et al., 1996). Again, however, this process is imperfect. Some environmental, cellular, or molecular factors can cause disruptions in this pathway, preventing the proper turnover of misfolded or unfolded proteins, potentially leading to their accumulation and aggregation. This generates a cellular condition known as ER stress (Friedlander et al., 2000; Walter and Ron, 2011; Karagöz et al., 2019).

Endoplasmic reticulum stress and the failure to correctly fold proteins are associated with loss of protein function and cell death (Zinszner et al., 1998; Hetz et al., 2006; Upton et al., 2012). To avoid this, the cell resolves misfolded protein stress via two major stress response pathways: the heat shock response (HSR) (Verghese et al., 2012), which handles misfolded proteins in the cytoplasm, and the unfolded protein response (UPR), which takes place in the ER (Kohno et al., 1993; Cox and Walter, 1996; Liu and Chang, 2008). These protein quality control mechanisms are essential for maintaining the function and integrity of cellular processes. When perturbed, they can lead to whole-cell dysfunction and toxicity (Ruis and Schüller, 1995; Voellmy, 2004). Under normal conditions, both lead to resolution of the cellular stress caused by the presence of misfolded proteins. In some cases, such as in several misfolded protein-associated diseases (Yoshida, 2007; Torres et al., 2015), these stress response pathways themselves can become impaired. This leads to further accumulation of misfolded proteins, which in turn causes further UPR or HSR impairment (Delépine et al., 2000; Zhang et al., 2002). Misfolded protein aggregates have also been shown to bind and sequester machinery important for degrading misfolded proteins via ER-associated degradation (ERAD), a protein quality-control mechanism which recognizes unfolded or misfolded proteins synthesized in the ER (Lippincott-Schwartz et al., 1988; McCracken and Brodsky, 1996). This ERAD impairment induces further stress in the ER and causes induction of the UPR. Proteostatic dysfunction essentially leads to a vicious cycle of increasing ER stress, protein accumulation, and stress response impairment.

The UPR is a complicated signaling pathway which works to resolve ER stress and allow protein synthesis and folding to continue and has been shown to interact with multiple cellular pathways and processes to do so, including (but not limited to) those occurring in the ER (Welihinda et al., 1999; Travers et al., 2000; Walter and Ron, 2011; Snapp, 2012). It has also been shown to be impacted by several seemingly unrelated external influences, including aging and lipid metabolism, and dysfunction in this pathway has been linked with shortened cellular lifespan and cell death (Jazwinski, 2002; Hou et al., 2014; Labunskyy et al., 2014). Because of this, the study of the molecular mechanisms behind ER stress and the UPR is essential to the understanding of how protein homeostasis impacts the entire cell and its processes, including response to stressors, aging, and cell death.

## Activation of the Unfolded Protein Response

As previously mentioned, the UPR is a stress response pathway specifically activated in response to ER stress, which is a condition that can be generated by things such as small molecules, environmental factors, or the accumulation of misfolded proteins in the ER (Welihinda et al., 1999). The UPR is activated when ER stress sensors embedded in the ER membrane detect the stressors and respond. Interestingly, the ultimate function of the UPR depends on the degree of activation and the length of time before the stress is resolved (Rutkowski et al., 2006; Rutkowski and Kaufman, 2007; Vidal and Hetz, 2012). It is primarily an adaptive response, which rescues cells from ER stress, but prolonged ER stress or high amplitude of UPR signaling causes the response to become maladaptive. In these circumstances, the UPR can activate alternate signaling pathways that result in apoptosis (Hetz et al., 2006; Rutkowski et al., 2006; Lin et al., 2007; Upton et al., 2012; Lu et al., 2014; Hetz and Papa, 2018).

In mammals, three distinct ER stress sensors exist: inositol requiring kinase 1 (IRE1) (Sidrauski and Walter, 1997; Yoshida et al., 2001; Calfon et al., 2002), double-stranded RNA-activated protein kinase like endoplasmic reticulum kinase (PERK) (Harding et al., 2000), and activating transcription factor 6 (ATF6) (Yoshida et al., 1998). When the UPR is activated, ER chaperone proteins (such as BIP) dissociate from these sensors, allowing their activation which in turn activates downstream signaling pathways (Welihinda et al., 1999; Shen et al., 2002; Ma and Hendershot, 2004; Pincus et al., 2010). Effector proteins from each of the three pathways bind to UPR response element (UPRE) sequences in gene promoters. A cell may activate over 400 UPR target genes involved in responding to ER stress, such as chaperone proteins, ribosome biogenesis genes, ERAD effectors, and genes to expand the ER lumen (Welihinda et al., 1999; Ma and Hendershot, 2004; Aragón et al., 2009). Upregulation of such genes contributes to adapt the ER folding environment to the new misfolded protein burden.

Despite its name, the UPR can be activated by stresses unrelated to misfolded or unfolded proteins. In addition to increased misfolded protein burden, ER stress can be induced by environmental factors; glucose deprivation/caloric restriction, for example, has been shown to mildly induce ER stress (Kaeberlein et al., 2005; Goldberg et al., 2009). Lipid concentration and composition in cells or in the extracellular environment have also been shown to significantly impact ER stress and UPR induction (Pineau et al., 2009; Promlek et al., 2011; Thibault et al., 2012). There is evidence to suggest that the UPR sensors IRE1 and PERK can detect perturbations of ER membrane lipid composition, independently of their luminal sensing domains, through their transmembrane domain (Promlek et al., 2011; Volmer et al., 2013; Kono et al., 2017). Other studies have also shown that the UPR is highly involved in responding to perturbation of lipid homeostasis (Thibault et al., 2012) and controls lipid synthesis and ER membrane proliferation in response to various cell stresses (Bernales et al., 2006; Schuck et al., 2009). Thus, UPR activation in the absence of unfolded proteins, via perturbation in the lipid composition of ER membrane, represents another regulatory mechanism (Promlek et al., 2011; Lajoie et al., 2012; Snapp, 2012; Volmer et al., 2013; Volmer and Ron, 2015) which may be important for UPR activation in ER stress-associated diseases.

Macroautophagy (henceforth, “autophagy”) is another protein quality control process which relies heavily upon functional membrane dynamics and proper membrane lipid composition. It is a non-specific maintenance process by which protein aggregates and damaged, defective, or aging cellular contents and organelles are transported to the lysosome for degradation (Klionsky and Emr, 2000; Mizushima, 2007; Glick et al., 2010). The membrane around the cargo destined for degradation by autophagy, the autophagosome, is derived from the ER membrane [with apparent contributions from the mitochondrial and plasma membranes (Nascimbeni et al., 2017)], wherein autophagic cargo are degraded by lysosomal hydrolases (Dunn, 1990). Autophagy occurs at a basal level in cells, but can be strongly induced by nutrient deprivation due to autophagy’s role in nutrient conservation and starvation adaptation, using bulk degradation to replenish amino acid availability for other cellular functions (Munafó and Colombo, 2001; Mizushima, 2007). ER stress has been shown to trigger autophagy, indicating parallel proteostatic responses to ER stress in the form of the UPR and autophagy (Bernales et al., 2006; Yorimitsu et al., 2006; Vidal and Hetz, 2012; Hou and Taubert, 2014). Importantly, activation of autophagy has been shown to be important for maintenance of proteostasis and lifespan regulation in multiple organisms and experimental models (Meléndez et al., 2003; Alvers et al., 2009a; Lee et al., 2012; Carroll et al., 2013).

## Er Stress and Aging

**FIGURE 1:**
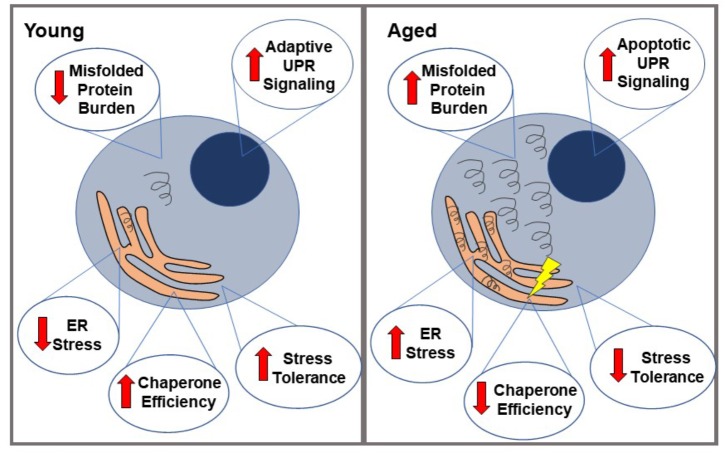
Proteostatic changes in aged vs. young cells. When faced with misfolded proteins, young cells demonstrate relatively low ER stress, high chaperone efficiency and stress tolerance, and primarily adaptive UPR signaling. This generally leads to a resolution of the misfolded proteins and therefore, ER stress. In contrast, aged cells are more likely to accumulate these misfolded proteins (partially due to loss of chaperone protein efficiency), leading to a state of ER stress, which they are less able to resolve. Their lower stress tolerance eventually leads to a relative increase in apoptotic UPR signaling over adaptive, ultimately causing cell death.

Aging has been shown to modulate some of the factors leading to ER stress. It is an important modifier of the proteostasis network, meaning that aging cells may have altered capacity to properly carry out protein transcription, translation, folding, and degradation (Naidoo, 2009; Brown and Naidoo, 2012; Figure 1). Aging cells have been shown to have decreased total levels of a number of ER proteins, including protein chaperones (such as PDI, BIP, etc.) which normally supervise and ensure proper protein folding, and assist in targeting misfolded proteins for degradation (Paz Gavilán et al., 2006; Hussain and Ramaiah, 2007; Naidoo et al., 2008). This usually prevents the accumulation and aggregation of misfolded proteins and prevents them from having toxic effects on the cell. In addition, the limited chaperones that are still present in the aging ER appear to be impaired. This is possibly due to an increased rate of oxidation of these chaperones in aged cells, leading to structural changes and consequently decreased function (van der Vlies et al., 2003; Snapp et al., 2006; Naidoo et al., 2008). For example, both BIP ATPase activity and PDI enzymatic function have been shown to be significantly decreased in aged mouse livers (Nuss et al., 2008), and similar results have been seen in a number of other models as well, such as aged mouse cerebral cortex (Naidoo et al., 2008). Other components of UPR signaling have also shown to be reduced during aging. PERK mRNA, for example, has been shown to be reduced in aged rat hippocampi, indicating less efficient UPR signaling (Paz Gavilán et al., 2006).

Aging also appears to alter the threshold at which the UPR switches from the adaptive pathway to the apoptotic pathway, which is perhaps related to the changes to proteostasis previously mentioned. When PERK signaling is decreased during aging, for example, there is evidence of an increase in GADD34 expression, which helps remove the translational block that occurs through PERK phosphorylating eIF2. This allows the expression of pro-apoptotic proteins, such as CHOP (Brown and Naidoo, 2012). CHOP has been shown to be increased with stress during aging and at baseline in aged muscular tissue in rats (Hussain and Ramaiah, 2007; Naidoo et al., 2008; Baehr et al., 2016); caspase-12 is also increased with stress in aged cells, but not during stress in younger cells (Paz Gavilán et al., 2006). The apoptotic protein JNK (which is activated by IRE1 during prolonged UPR signaling) is also upregulated during aging, as are JNK kinases that phosphorylate other apoptotic transcription factors such as ATF-2 and c-Jun (Hussain and Ramaiah, 2007; Brown and Naidoo, 2012). Calcium-mediated cell death pathways are also altered during aging. Aging has been linked to increased calcium flux between the ER and mitochondria, and consequently increased exposure to reactive oxygen species and sensitivity to cell death in the case of mitochondrial calcium overload (Fernandez-Sanz et al., 2014; Calvo-Rodríguez et al., 2016; Madreiter-Sokolowski et al., 2019). These factors, in turn, lead to a decreased threshold for the activation of calcium-mediated apoptosis. This aging-related decrease in adaptive UPR signaling and increase in apoptotic signaling may account for the apparent sensitivity of aged cells to ER stress, and the increased rate of cell death amongst stressed cells when aged (Rutkowski et al., 2006; Lu et al., 2014; Tay et al., 2014). Autophagy has also been shown to become less efficient at clearing damaged organelles and misfolded proteins during aging in yeast (as well as other models), and lifespan extension has been demonstrated when autophagy is heavily induced (Zhang and Cuervo, 2008; Alvers et al., 2009b; Caramés et al., 2010; Koga and Cuervo, 2011; Martinez-Lopez et al., 2015).

## Er Stress and Age-Dependent Human Diseases

Aging is a common risk factor for a number of protein misfolding diseases, including several neurodegenerative diseases (Martínez et al., 2017), a number of which have links to UPR function as described in the previous section. While not all aging-related diseases are directly linked to breakdown of UPR signaling, this breakdown may still contribute to disease pathogeneses. For example, type 2 diabetes is known to develop more frequently due to both obesity and aging, with the two factors often coexisting in patients (Ozcan et al., 2004). As aging has also been linked to the decreases in UPR effector proteins associated with diabetes and insulin resistance, these results suggest that aging-related UPR defects may be linked to these diseases as well. Obesity and insulin resistance are also linked to heart disease and atherosclerosis, both of which increase in prevalence with age and have also been linked to ER stress and the UPR (Han et al., 2006). Huntington’s disease (HD), Parkinson’s disease, and Alzheimer’s disease have been clearly and repeatedly linked to UPR dysfunction which increases with age, thus increasing disease severity (Vidal and Hetz, 2012; Carroll et al., 2013). Though the three diseases have different causative genes, they all share misfolded protein accumulation and aggregation as part of their pathology, leading to impaired proteostasis and ER stress responses, and then cellular toxicity.

In Alzheimer’s disease, for example, tau neurofibrillary tangles and amyloid-β plaques accumulate in neurons and lead to neurodegeneration (Lee et al., 2010). Studies have shown that cells with these protein aggregates have high UPR induction identified through high levels of phosphorylated eIF2α, PERK, and IRE1 (Hoozemans et al., 2005, 2009; Gerakis and Hetz, 2018a,b). This has been identified in early stages of protein accumulation and linked to later stage neurodegeneration, suggesting an early beneficial role for the UPR that may later become maladaptive (Hoozemans et al., 2009). Indeed, as cells age, the UPR’s capacity to cope with the misfolded protein load decreases in an fly model of Alzheimer’s disease; decreased signaling through the IRE1 branch of the UPR has been identified in this model, leading to decreased misfolded protein clearance (Marcora et al., 2017). Similar findings have also been reported in spinal cord tissue from patients with sporadic Amyotrophic Lateral Sclerosis (ALS) (Atkin et al., 2008). Enhancing UPR signaling and/or reducing ER stress through genetic and pharmacological modulation of UPR effectors such as eIF2α, PERK, XBP1, ATF4, and heat shock proteins have all been shown to have positive effects on various models of ALS (Hetz et al., 2009; Saxena et al., 2009; Castillo et al., 2013; Matus et al., 2013; Saxena et al., 2013; Jiang et al., 2014; Wang et al., 2014a,b; Das et al., 2015; Vieira et al., 2015; Nagy et al., 2016). Huntington’s disease is another neurodegenerative disease which is characterized by the accumulation of misfolded huntingtin protein, which undergoes abnormal expansion of a segment of polyQ repeats (Penney et al., 1997). Longer polyQ tracts are associated with earlier onset and more severe symptoms (Macdonald, 1993), and are also more prone to aggregation and are associated with a higher degree of UPR induction but a lower degree of HSR induction (Martindale et al., 1998; Chafekar and Duennwald, 2012). These aggregates have also been shown to cause ER stress and impaired ERAD due to sequestration of ERAD machinery, leading to UPR hyperactivation (Duennwald and Lindquist, 2008; Lajoie and Snapp, 2011; Leitman et al., 2013; Jiang et al., 2016). In agreement with dysregulated UPR in HD, restoration of “normal” XBP1 and PERK activity has been shown to improve disease phenotypes in both cell and animal models (Vidal et al., 2011, 2012; Leitman et al., 2014; Rivas et al., 2015). Importantly, activation of ER stress pathways has been detected in post-mortem patient samples (Carnemolla et al., 2009). Similarly, UPR has been associated with the onset of Parkinson’s disease (Mercado et al., 2016). Accumulation of α-synuclein has been shown to block ER to Golgi trafficking and consequently activate the UPR in both yeast and humans (Cooper et al., 2006; Heman-Ackah et al., 2017). PERK inhibition showed positive effects in a mouse model of Parkinson’s disease (Celardo et al., 2016; Mercado et al., 2018). In Parkinson’s disease, misfolded proteins accumulate in the substantia nigra region of the brain, leading to loss of dopaminergic neurons in this region. Similar to the studies performed on Alzheimer’s disease, it has been shown that the UPR is highly activated in these areas and that this UPR activation may be causally linked to the neurodegeneration seen in this disease (Hoozemans et al., 2007). ER stress is therefore a common determinant of multiple neurodegenerative diseases. Thus, targeting the UPR has emerged as an attractive therapeutic approach these disorders (Rivas et al., 2015; Valenzuela et al., 2016).

## Conclusion

As more connections are drawn between ER proteostasis and aging, it becomes clear that these interactions reach much further than previously thought – from neurodegeneration to temperature adaptation, and from simple model organisms like yeast up to higher mammals. Future research on these fields (individually and as a whole) will hopefully address some yet-unanswered questions on how and why these connections exist. For example, could age-related changes in membrane composition and fluidity explain age-related increases in UPR signaling? What advantage would be conferred to the cell by UPR stress-sensing proteins responding to changes in membrane lipids as well as ER stress? What other cellular functions and pathways intersect with these processes, in both baseline and stressed/aged states? In all likelihood, there is not one single cause for the breakdown of ER homeostasis during aging, but instead a combination of factors contributes to overall increased sensitivity to ER stress. Increased misfolded protein accumulation, decreased effectiveness of the adaptive UPR, and an altered threshold for apoptotic UPR signaling likely all play a role (Naidoo et al., 2008; Brown and Naidoo, 2012; Madreiter-Sokolowski et al., 2019). Finally, ER stress sensitivity is not only dictated by the amplitude of the UPR response but also by upregulation of a specific set of target genes required to adapt the ER folding environment for a given stress-causing situation (Thibault et al., 2011). Therefore, the question of what categories of UPR target genes define the aging UPR is a crucial one that needs to be addressed. As technology and research methods advance and our understanding of these areas improves, this future research will likely have important implications for basic science and therapeutic approaches to human diseases.

## Author Contributions

PL and SC wrote the manuscript. SC generated the figure.

## Conflict of Interest Statement

The authors declare that the research was conducted in the absence of any commercial or financial relationships that could be construed as a potential conflict of interest.
